# Preparing the public health workforce for digital engagement: early evaluation of a massive open online course on using social media for health promotion

**DOI:** 10.3389/frhs.2026.1960826

**Published:** 2026-10-07

**Authors:** Kahlia McCausland, Tama Leaver, Katharina Wolf, Becky Freeman, Gwyneth Peaty, Justine E. Leavy, HuiJun Chih, Barbara A. Mullan, Sonya Girdler, Mark Chenery, Jonine Jancey

**Affiliations:** 1 Collaboration for Evidence, Research and Impact in Public Health, School of Population Health, Curtin University, Bentley, WA, Australia; 2 School of Media, Creative Arts and Social Inquiry, Curtin University, Bentley, WA, Australia; 3 School of Management and Marketing, Curtin University, Bentley, WA, Australia; 4 School of Public Health, University of Sydney, Sydney, NSW, Australia; 5 Australian Centre for Student Equity and Success, Curtin University, Bentley, WA, Australia; 6 School of Population Health, Curtin University, Bentley, WA, Australia; 7 Common Cause Australia, Canberra, ACT, Australia

**Keywords:** digital health, health promotion, massive open online course (MOOC), public health communication, social media, workforce development and training

## Abstract

**Introduction:**

Health professionals are increasingly expected to engage effectively in digital environments; however, many lack any training in digital communication and social media use. This paper reports findings from the early evaluation of a Massive Open Online Course (MOOC) designed to strengthen public health workforce capability in digital communication and advocacy.

**Methods:**

Quantitative and open-ended survey responses relating to perceived relevance, learning outcomes, usefulness of course components, and early application in professional practice were analysed from interim post-test (T1) and post-test (T2) surveys conducted as part of an ongoing evaluation embedded within a wait-list randomised controlled trial. Quantitative data were analysed descriptively, while open-ended responses were analysed using inductive thematic analysis.

**Results:**

A total of 101 Australian public health and health promotion professionals contributed data to the T1 survey and 60 contributed to the T2 survey. Participants reported high levels of perceived relevance (88.5%) and knowledge gain (86.5%) following course completion (T1). At T2, 72.9% reported a positive impact on professional practice, although fewer reported practice changes (51.7%) or direct application of learning (56.7%). Videos, visuals, and case studies were the most highly rated learning resources, while the discussion board was the least useful. Qualitative findings identified message framing, values-based communication, mixed media delivery, expert-led content, and applied examples as key facilitators of learning. Participants described applying learning to communication, advocacy, and social media activities. Suggested improvements included greater practical depth, enhanced interactivity, and improved platform functionality.

**Conclusion:**

Early findings suggest that a flexible, online MOOC can support the development of digital communication capabilities among public health professionals. While participants reported substantial learning gains, stronger opportunities for applied learning, interaction, and practice may be required to support translation of learning into sustained professional practice.

## 1 Introduction

Digital technologies, including social media platforms and other digitally mediated communication tools, play a central role in health promotion, public health communication, advocacy, and the delivery of virtual health services (1, 2). Health professionals increasingly use digital channels to disseminate evidence-based information, engage communities, counter mis/disinformation, and influence policy and practice (3, 4). Consequently, preparing the public health workforce to engage effectively in these digital environments has become an essential professional competency (5). Workforce preparedness requires more than access to technology; it also requires structured, contextually relevant training that builds skills, confidence, and critical understanding of digital platforms, audience engagement, and the ethical and practical challenges associated with communicating online. These capabilities align with contemporary public health and health promotion priorities that increasingly emphasise digitally enabled communication, community engagement, and population-level behaviour change (2, 5, 6).

The need for these competencies is reinforced by the contemporary digital information environment, where commercial actors increasingly shape public discourse and health behaviours (7) through targeted advertising, influencer partnerships, and algorithm-driven content (8, 9). This reflects the commercial determinants of health, defined as the systems, practices, and pathways through which commercial actors influence health and equity (10). The World Health Organization (11) notes that these influences extend beyond marketing to include mis/disinformation and lobbying that are used to protect commercial interests, often at the expense of population health. Industries producing unhealthy commodities, including tobacco, e-cigarettes, alcohol, gambling, and ultra-processed foods, have become particularly effective at using digital platforms to normalise product use and challenge public health messaging (12). As a result, health professionals require both technical digital communication skills and the ability to recognise and respond to commercial influences operating within online environments.

There is growing recognition that many health professionals lack training in digital engagement (13–15). However, Australian evidence has been limited (16). To address this gap, we conducted a national survey of public health and health promotion professionals (17). Although most respondents used social media in their professional roles, formal training was uncommon, and many respondents rated their social media competency as basic or intermediate. Importantly, participants reported significant concerns about professional social media use, including the validity and quality of information online, exposure to mis/disinformation, inappropriate online behaviour (e.g., trolling and harassment), privacy, and personal safety. The findings informed the development of a Massive Open Online Course (MOOC) (18), designed to prepare the public health workforce for the ethical, strategic, and effective use of digital and social media in health promotion, communication and advocacy.

This paper reports preliminary findings from the evaluation of the MOOC, focusing on interim post-test (T1) and post-test (T2) survey data. Specifically, we present quantitative and qualitative results on participants' perceptions of relevance, learning outcomes, the usefulness of course components, and early examples of application in professional practice. These findings provide insight into how flexible online training can support workforce capability in digital health communication and advocacy. The full evaluation, including the randomised controlled trial (RCT) outcomes, will be reported in a separate paper.

## 2 MOOC development

MOOCs are structured online courses designed to provide flexible, self-paced learning for large, geographically dispersed cohorts. In public health and health workforce education, they offer a flexible and accessible approach to continuing professional development, particularly where learners are distributed across locations, need timely access to knowledge and must balance study with professional commitments (19, 20).

### 2.1 Aim

In response to the identified training gaps and the need for digital knowledge and skills for application to real-world problems, a team of subject experts developed a six-module MOOC titled “*Using Social Media for Effective Health Promotion, Communication and Advocacy”*, now available globally via the edX platform (21) (and accessible here: https://www.edx.org/learn/health-safety/curtin-university-social-media-messaging-in-health-promotion).

The MOOC aims to improve public health and health promotion professionals' knowledge, skills, and confidence in using social media as a tool for health promotion, communication, advocacy and digital engagement. Specifically, the MOOC seeks to:

- build a foundational understanding of social media as a digital health communication and advocacy tool;
- strengthen capabilities in framing health messages for diverse audiences;
- enhance confidence in professional digital interactions and engagement;
- equip learners to recognise and respond appropriately to misinformation, disinformation, and online opposition;
- strengthen learners’ ability to identify, critically appraise, and respond to commercial influence and digital marketing strategies that undermine public health; and
- support ethical, strategic, and sustainable use of social media in professional practice.

### 2.2 Development process and pedagogical model

The MOOC was developed over 16 months by an interdisciplinary team of Australian-based academics and practitioners from public health, health promotion, communication, psychology, media studies, and social science. This team's diverse composition was deliberate, aiming to reflect the multifaceted nature of digital engagement in health and to integrate theoretical, empirical, and practice-based perspectives, while also ensuring it is interactive, relevant and engaging.

The course follows an xMOOC pedagogical model (22), characterised by structured content delivery, short lecture-style videos, applied case studies, readings, quizzes, and reflective activities. This format supports accessible, flexible and self-paced learning, while maintaining academic rigour and practical relevance for working professionals seeking modular training that can be integrated into ongoing professional development. This approach also accommodates different learning styles, offering a range of ways to access and process new information.

Each module went through multiple rounds of drafting and review to ensure clarity, relevance, and alignment with learning objectives. The first draft for each module was created by a single subject expert (JJ, TL, BF, MC, KW) in collaboration with the team's learning designer (GP), who has experience in curriculum development and online training formats. This allowed the content to foreground individual specialist knowledge in each subject area while still ensuring stylistic and pedagogical consistency across the modules. Later drafts were shared with the broader academic partnership for feedback and refinement, ensuring that each module benefited from the combined expertise of the team.

The course was designed to be accessible to learners with varying levels of experience with social media and digital technologies. Content did not assume prior knowledge; instead, key concepts and terminology were introduced and explained as they arose. To support the translation of theory into practice, modules incorporated real-world case studies focused on the commercial determinants of health, particularly tobacco and vaping industry marketing tactics and public health responses. These examples were used to illustrate how theoretical concepts relate to digital communication and advocacy challenges commonly encountered in professional practice. Visual and interactive elements were incorporated throughout the course to clarify concepts, reinforce key messages, and reduce reliance on extended written text. Subject experts were featured as module hosts, presenting video content within their areas of expertise to provide continuity across the course and strengthen the connection between learners and the content. The final module concludes with a panel discussion involving expert presenters, providing an opportunity to synthesise key learning and explore future challenges and opportunities in digital health communication.

### 2.3 Overview of MOOC content

The MOOC comprises six modules, each building progressively on the previous content:

1. **Exploring the Commercial Determinants of Health:** Introduces the concept of commercial determinants of health and their influence on public health, using unhealthy commodities (e.g., tobacco and e-cigarettes) as a recurring case study to situate digital engagement within broader political, economic, and commercial contexts.
2. **Social Media Essentials:** Covers the evolution of social media, key platform features, content types, algorithms, the shift from one-to-many to many-to-many communication, and the dominance of visual social media, establishing foundational digital literacies.
3. **Using Social Media for Public Health Education and Advocacy:** Explores the strategic uses of social media for education, communication and advocacy, drawing on real-world examples of tobacco and vaping campaigns, policy influence, and public discourse.
4. **Message Framing:** Discusses how message framing influences audience interpretation and engagement, with practical guidance for designing effective health messages.
5. **Social Media Pitfalls and Challenges:** Discusses mis/disinformation, online harassment, trolling, and reputational risk, and provides practical response strategies.
6. **The Future of Health Promotion on Social Media:** Features a panel discussion with subject experts reflecting on emerging trends, opportunities and challenges in digital health promotion.

## 3 Preliminary methods

### 3.1 Study design and procedure

This study forms part of a larger mixed-methods evaluation of a MOOC, embedded within a wait-list RCT (18), using relevant data collected via online surveys at interim post-test (T1), immediately following MOOC delivery; and post-test (T2), approximately four months following MOOC delivery.

Australian health promotion and public health professionals were recruited through organisations represented on the Project Advisory Group, as well as through professional networks and relevant agencies contacted directly by the research team. Recruitment materials were distributed to members and employees via email, newsletters, and social media, and were further promoted through study-specific social media activities. Interested individuals completed an online expression of interest form in Qualtrics to determine eligibility. Participants were required to: (a) be aged 18 years or older; (b) reside in Australia; (c) be employed in a public health and/or health promotion role; and (d) have no financial or professional conflict of interest with organisations involved in the production, distribution, or sale of tobacco, nicotine, or vaping products.

Participants were invited to complete each survey via email. At the start of each survey, participants were given a Participant Information Statement and asked to provide informed consent. The present analysis reports preliminary findings related to learner reactions, perceived learning, and early self-reported application of knowledge, aligning with selected domains of the RE-AIM (23) and Kirkpatrick (24) evaluation frameworks.

Further details regarding the study design and evaluation procedures are reported elsewhere (18). The trial is registered with ClinicalTrials.gov and the Australian New Zealand Clinical Trials Registry (ACTRN12624001486516). Ethical approval was granted by the Curtin University Human Research Ethics Committee (HRE2023-0020).

### 3.2 Survey instrument

The online Qualtrics survey included categorical, ordinal, and open-ended questions. The survey assessed participants’ social media experience, confidence and competency in public health communication and advocacy, perceived knowledge and skills, experiences of course completion, perceived impact on professional practice, and satisfaction with the MOOC. Items measured agreement, confidence, comfort, experience, usefulness, or frequency of application. Participants also rated the usefulness of specific course components (videos, case studies, activities, quizzes, and discussion board). Open-ended questions asked participants to describe key learning outcomes, examples of professional application, the most and least useful aspects of the course, and suggestions for improvement.

### 3.3 Quantitative analysis

Quantitative data were analysed using IBM SPSS Statistics (Version 31.0). Descriptive statistics were calculated for all survey items and are presented as frequencies and percentages. For ease of interpretation, responses to two five-point Likert scales (strongly agree, somewhat agree, neither agree nor disagree, somewhat disagree, strongly disagree) were collapsed into three categories: agree (strongly agree/somewhat agree), neither agree nor disagree, and disagree (somewhat disagree/strongly disagree). Responses to helpfulness items (very helpful, helpful, somewhat helpful, not particularly helpful, not at all helpful) were collapsed into two categories: helpful (very helpful/helpful/somewhat helpful) and not helpful (not particularly helpful/not at all helpful).

As response categories were derived from ordinal (Likert-type) items, each category was treated as an individual binomial proportion. Ninety-five percent confidence intervals were calculated using the Wilson score method, which provides more accurate interval estimates than the normal approximation method, particularly for smaller sample sizes and proportions close to 0 or 100% (25). Analyses were conducted separately for the T1 and T2 surveys, with the number of responses received for each item reported to illustrate variation in response rates.

### 3.4 Qualitative analysis

Open-ended survey responses collected at T1 and T2 were analysed using inductive thematic analysis (26). Responses were first reviewed to establish familiarity with the data and then imported into NVivo (Version 15) for coding. Initial codes were developed directly from participants' comments and grouped into broader categories based on recurring ideas and patterns. Related categories were subsequently organised into themes that captured common experiences, perceptions, and suggestions across responses. No *a priori* coding framework was applied, and themes were derived inductively from the dataset. Results are presented as thematic summaries supported by illustrative participant quotes.

The lead author, who conducted the interviews and analysis, was not involved in the design, development, or delivery of the MOOC and undertook the analysis independently of the course development team. As researchers working in public health and health promotion, we acknowledge that our professional experience and familiarity with digital health education may have influenced the interpretation and emphasis placed on particular findings. We sought to minimise this influence by reporting findings transparently, including those identifying limitations of the MOOC.

## 4 Results

Participant characteristics are reported in Table 1. Due to attrition, intermittent non-response, and incomplete linkage of survey responses across time points, the analytic sample did not constitute a complete longitudinal cohort. Analyses were therefore based on all available responses at each time point, and sample sizes vary. A total of 101 participants contributed data to the T1 survey, and 60 contributed data to the T2 survey.

**Table 1:** Characteristics of participants included in the post-test analyses (*n* = 102).

Characteristic	Response option	*n* (%)
Age (years)	18–29	30 (29.4)
	30–39	26 (25.5)
	40–49	25 (24.5)
	50–59	15 (14.7)
	60+	6 (5.9)
Gender	Female	92 (90.2)
	Male	9 (8.8)
	Non-binary	1 (1.0)
Educational attainment	Postgraduate qualification	99 (97.1)
	Vocational Education and Training	3 (2.9)
Role	Practitioner	63 (61.8)
	Policymaker	11 (10.8)
	Researcher/academic	28 (27.5)
Years of experience in public health	<2 years	27 (26.5)
	>2–5 years	23 (22.5)
	>5–10 years	21 (20.6)
	>10–25 years	23 (22.5)
	>25 years	8 (7.8)
Previous social media training	Yes	10 (9.8)

Results are presented in two sections: (i) quantitative survey findings relating to perceived relevance, learning outcomes, and resource usefulness, and (ii) qualitative survey findings describing participant experiences of the MOOC, including facilitators and barriers to engagement and application. Findings relating to participants' experiences of the MOOC were drawn from the T1 survey, whereas findings relating to application in professional practice were drawn from the T2 survey to allow time for learning to be applied in workplace settings.

### 4.1 Participant characteristics

Participant characteristics are reported in Table 1. Most participants were aged 18–29 years (29.4%), followed by 30–39 years (25.5%) and 40–49 years (24.5%). The sample was predominantly female (90.2%) and highly educated, with most participants holding a postgraduate qualification (97.1%). Only 9.8% of participants had previous social media training.

### 4.2 Quantitative results

#### 4.2.1 Perceived relevance, knowledge gain, and helpfulness of MOOC resources

Perceived relevance and learning outcomes were consistently high at T1 (Table 2), with close to nine out of ten respondents agreeing that the content was relevant to their professional practice (88.5%) and that they had gained new knowledge and skills (86.5%). Perceived helpfulness varied by resource type. Visuals (97.8%), embedded videos (96.7%) and case studies (96.7%) received the strongest ratings. Written content (93.4%) and activities (93.2%) were also well regarded. In contrast, interactive and social components were less well received. The discussion board stood out as the least useful element, with 58.0% rating it as not helpful. The panel discussion and external video content elicited mixed responses, with higher proportions of neutral or lower-helpfulness ratings. Confidence intervals were generally narrow for T1 estimates, indicating relatively precise estimates for most items.

**Table 2:** Perceived relevance, knowledge gain, and helpfulness of MOOC resources at T1 (*n* = 101).

Survey item	Response option	*n* (%)	95% CI
The content delivered in the MOOC was relevant to my professional practice (*n* = 96)	Agree	85 (88.5)	80.6%–93.5%
	Neither agree nor disagree	8 (8.3)	4.3%–15.6%
	Disagree	3 (3.1)	1.1%–8.8%
I have gained new knowledge and skills as a result of taking the MOOC (*n* = 96)	Agree	83 (86.5)	78.2%–91.9%
	Neither agree nor disagree	6 (6.3)	2.9%–13.0%
	Disagree	7 (7.3)	3.6%–14.3%
Perceived helpfulness of written content (*n* = 91)	Helpful	85 (93.4)	86.4%–96.9%
	Not helpful	6 (6.6)	-
Perceived helpfulness of case studies (*n* = 91)	Helpful	88 (96.7)	90.8%–98.9%
	Not helpful	3 (3.3)	-
Perceived helpfulness of embedded videos (*n* = 91)	Helpful	88 (96.7)	90.8%–98.9%
	Not helpful	3 (3.3)	-
Perceived helpfulness of YouTube/external videos (*n* = 90)	Helpful	81 (90.0)	82.1%–94.6%
	Not helpful	9 (10.0)	-
Perceived helpfulness of links to further readings/references (*n* = 89)	Helpful	77 (86.5)	77.9%–92.1%
	Not helpful	12 (13.5)	-
Perceived helpfulness of visuals (*n* = 91)	Helpful	89 (97.8)	92.3%–99.4%
	Not helpful	2 (2.2)	-
Perceived helpfulness of activities (*n* = 88)	Helpful	82 (93.2)	85.9%–96.8%
	Not helpful	6 (6.8)	-
Perceived helpfulness of discussion board (*n* = 69)	Helpful	29 (42.0)	31.1%–53.8%
	Not helpful	40 (58.0)	-
Perceived helpfulness of end of module quizzes (*n* = 90)	Helpful	80 (88.9)	80.7%–93.9%
	Not helpful	10 (11.1)	-
Perceived helpfulness of panel discussion (*n* = 80)	Helpful	60 (75.0)	64.5%–83.2%
	Not helpful	20 (25.0)	-

#### 4.2.2 Perceived impact of the MOOC on professional practice

At T2, reported impacts on practice were moderate and variable (Table 3). While the majority reported a positive overall impact (72.9%), far fewer reported translating this into practice change (51.7%) or applying learning (56.7%), with around one-third of respondents selecting neutral responses for both items. Sharing knowledge with colleagues was similarly mixed (55.0%), with nearly three in ten respondents reporting neither agreement nor disagreement. Wider confidence intervals were observed for some T2 outcomes, reflecting the smaller number of respondents at follow-up.

**Table 3:** Perceived impact of the MOOC on professional practice at T2 (*n* = 60).

Survey item	Response option	*n* (%)	95% CI
I have changed some aspect of my professional practice as a result of taking the MOOC (*n* = 60)	Agree	31 (51.7)	39.3%–63.8%
	Neither agree nor disagree	21 (35.0)	24.2%–47.6%
	Disagree	8 (13.3)	6.9%–24.2%
Taking the MOOC has had a positive impact on my professional practice (*n* = 59)	Agree	43 (72.9)	60.4%–82.6%
	Neither agree nor disagree	13 (22.0)	13.4%–34.1%
	Disagree	3 (5.1)	1.7%–13.9%
I have shared what I have learned with colleagues (*n* = 60)	Agree	33 (55.0)	42.5%–66.9%
	Neither agree nor disagree	17 (28.3)	18.5%–40.8%
	Disagree	10 (16.7)	9.3%–28.0%
I have applied what I learned from completing the MOOC in my professional practice (*n* = 60)	Agree	34 (56.7)	44.1%–68.4%
	Neither agree nor disagree	19 (31.7)	21.3%–44.2%
	Disagree	7 (11.7)	5.8%–22.2%

### 4.3 Qualitative results

The findings are presented in two parts: facilitators (features that supported engagement and learning) and barriers (areas identified for improvement).

#### 4.3.1 Facilitators: what was done well

Participants identified several features that supported engagement, learning, and early application of content within professional practice.

##### 4.3.1.1 Strategic communication skills

Participants consistently identified message framing and values-based messaging as core strengths of the MOOC, particularly in relation to their applicability to professional communication tasks. Learning in this area was described as transferable across formats, including written, verbal, and digital communication. Participants reported using these approaches in practice, for example, “*applying values-based messaging to posters, social media, and articles”* and “*using message framing when drafting speaking notes.”* Some explicitly linked this learning to improved communication effectiveness, describing a greater capacity to deliver “*the right messages to the right audiences in the right way.”*

##### 4.3.1.2 Flexible mixed media delivery

The use of varied content formats was consistently described as supporting engagement. Participants highlighted the integration of text, video, and interactive elements, noting that “*the blend of text, video, interactive elements and imagery … kept the content interesting”* and that “*none of the content (written or video) felt exhausting or tedious to get through.”* Flexibility in delivery was also valued, with participants reporting that they could engage “*at my own pace”* and “*dip in and out”* of the course within existing work and family commitments.

##### 4.3.1.3 Expert-led content and credibility

Participants emphasised the role of expert presenters in supporting learning. Exposure to subject matter experts was described as enhancing credibility and relevance, with participants noting “*having industry experts provide up-to-date information”* and that “*the videos from the various experts were excellent.”* The inclusion of multiple perspectives was also highlighted, with participants reflecting that “*it was great to have a variety of experts.”*

##### 4.3.1.4 Applied learning through case studies and examples

Case studies and real-world examples were identified as facilitating understanding and application. Participants described these elements as helping translate theory into practice, noting that examples “*weren't just theory-based”* and that “*case studies and examples help to clarify the messages.”* Exposure to both effective and ineffective examples, including “*examples from others (successful and not)”* and “*real-world design examples and experiences”* was also highlighted as useful.

##### 4.3.1.5 Reinforcement of learning

Supplementary resources, including summaries, additional readings, and downloadable materials, were described as supporting learning and retention. Participants referred to “*the resources including the end of module summary, and the list of additional reading”* and “*downloadable tip sheets”* that could be revisited later. Quizzes were also described as reinforcing learning, with participants noting they were “*a great way … to retain my information.”* Accessibility features, such as transcripts, supported engagement with different learning preferences.

##### 4.3.1.6 Application to professional practice

Participants described applying their learning across a range of professional contexts, particularly in digital communication. This included developing and refining campaigns, such as “*develop[ing] a LinkedIn campaign to promote recent policy work,”* as well as using learning to “*map out and develop social media campaigns.”* Participants also reported improved collaboration with communication teams, including “*better collaborat[ing] with internal and external marketing and comm[unications]s professionals,”* and sharing knowledge through activities such as “*deliver[ing] workshops to health promotion colleagues.”*

#### 4.3.2 Barriers: what could be improved

Participants identified a range of areas where the MOOC could be strengthened, spanning content depth, opportunities for interaction, and aspects of delivery and usability.

##### 4.3.2.1 Content depth and alignment with professional needs

Some participants identified the level of content as a key area for improvement, particularly regarding alignment with professional audiences. Some described the content as too broad or introductory, noting it was “*tailored more toward the general public rather than health professionals”* and that “*the information was way too simple.”* Participants with prior experience expressed a preference for more detailed and advanced content, including “*more granular, practical information.”*

##### 4.3.2.2 Limited practical depth and ‘how-to’ guidance

While conceptual material was valued, some participants reported a need for more detailed guidance on implementation. This included requests for “*real grounded experience on the ‘how to’”* and clearer direction on “*what specific strategies we can deploy.”* Some participants also suggested including more applied examples and exercises, such as “*more specific examples of good v bad social media content.”*

##### 4.3.2.3 Course structure, pacing, and workload

Some participants highlighted aspects of the course structure and workload as limiting engagement. Several noted that “*modules took a lot longer than I anticipated,”* and suggested structural changes, including to “*condense some of the content”* or “*consider extending course duration.”*

##### 4.3.2.4 Engagement features and interaction design

Engagement features, particularly the discussion board, were consistently described as under utilised. Participants reported that they “*didn't engage with this beyond the introductions”* and that it was “*hard to engage with others as everyone is at different stages.”* The asynchronous format was identified as a barrier, with participants noting that the discussion board “*wasn't in real time”* and that “*not many people had posted.”* Suggestions for improvement included increasing the discussion board's visibility within modules and introducing more structured or moderated interaction.

##### 4.3.2.5 Platform usability and technical issues

Technical issues and usability challenges were identified as affecting engagement for some participants. One participant reported “*issues with the videos loading”,* and another said that the progress tracking feature “*did not capture that a section had been completed.”* Difficulties with navigation and access were also described by a couple of participants, with one participant suggesting the need to “*find a new platform to host content.”*

##### 4.3.2.6 Assessment and supporting resources

Some participants identified assessment and resource integration as areas for refinement. Some described the end of module quizzes as too simple, and suggestions were made to redesign them to better support learning. Participants also expressed a preference for better integration of external materials, indicating that resources could be more effectively embedded within the course rather than provided as standalone links.

## 5 Discussion

The digital transformation of public communication has created new demands on the public health and health promotion workforce (1). Professionals are increasingly expected to engage with digital platforms strategically, credibly, and safely, yet the competencies required for effective professional digital engagement and public-facing communication remain a significant gap in workforce development (13, 14). This interdisciplinary work responds directly to that need by providing early evidence from the evaluation of a MOOC designed to support the public health workforce in digital health promotion, communication, and advocacy.

Despite the high educational attainment of participants, formal training in digital communication appeared uncommon. Nearly all participants held a postgraduate qualification (97.1%), yet fewer than one in ten reported having received previous social media training. This finding suggests that competency in digital communication and social media engagement is not routinely developed through traditional public health or health promotion education pathways. Similar gaps have been reported internationally, where widespread professional use of social media has not been matched by formal training opportunities or established competency-based curricula (13). This is particularly notable given growing expectations that health professionals communicate effectively in digital environments, counter mis/disinformation, engage communities online, and navigate platform-specific communication challenges. Recent Australian evidence suggests that the digital capability of the health workforce has not kept pace with the increasing integration of digital technologies into healthcare and public health practice (27). Systematic review evidence similarly identifies insufficient training and organisational support as key barriers to digital readiness and capability among health professionals and recommends integrating specialised digital training into workforce development initiatives (28). The findings suggest opportunities for universities, professional associations, and employers to embed digital communication training within formal education, continuing professional development, micro-credentials, and workplace-based learning initiatives.

Participants reported high perceived relevance and substantial knowledge gain, while qualitative findings demonstrated acquisition of competencies related to message framing, values-based communication, audience engagement, and understanding digital platforms. These capabilities align with competency frameworks that identify strategic communication, digital professionalism, and effective use of social media as core skills for health professionals working in digital environments (1, 5, 13). These findings suggest that participants perceived the MOOC as addressing competency areas that are increasingly recognised as central to effective digital health communication practice.

Participants particularly valued learning that was clearly applicable to practice, with message framing and values-based communication consistently identified as the most useful components. This aligns with the need for health professionals to move beyond information provision towards more strategic communication approaches in digital spaces, where message reach and engagement are shaped by both platform features and audience alignment (29, 30). Participants also reported increased understanding of how platform dynamics and commercial actors influence health communication, pointing to the importance of systems-level knowledge as part of workforce preparation for digital health (1). Collectively, these findings suggest that the MOOC contributed to building foundational capabilities needed for effective communication, advocacy, and engagement in increasingly digital public health practice environments.

The role of delivery design was also evident. High ratings for videos, visuals, and case studies, alongside qualitative feedback on mixed media and flexible access, indicate that mode of delivery played a key role in supporting engagement. Participants described the combination of formats as maintaining interest and allowing learning to fit within work and family contexts. This is consistent with broader evidence that flexible, asynchronous models are well-suited to professional learning in digital health, where competing demands on time are common (31, 32). Expert presenters and the multidisciplinary teaching team appeared to strengthen credibility and relevance by exposing learners to diverse perspectives and real-world experiences in digital communication, advocacy, and public engagement. Consistent with contemporary guidance on quality online course design, these findings suggest that learner engagement is influenced not only by course content, but also by the deliberate integration of multimedia resources, practical learning activities, diverse perspectives, and accessible course structures that support participation and knowledge retention (33).

Despite strong reported learning, evidence of behavioural change was mixed. While most participants reported a positive impact on practice, fewer described actual changes to professional practice or consistent application of learning four months following MOOC delivery. Qualitative findings help provide insight into this gap. Some participants described applying concepts directly, such as developing campaigns, adapting messaging, and influencing organisational communication strategies. Others reported indirect application, including increased awareness, critical reflection, and changes in how they approached online engagement. However, several participants noted limited opportunity to apply learning within their current roles or described needing more practical guidance to translate concepts into action. This gap between knowledge acquisition and application reflects a broader challenge in digital health workforce development (34). Evidence from online professional learning programs consistently demonstrates improvements in knowledge, skills, and confidence (32); however, translation of learning into practice is often less evident and may require additional implementation supports, opportunities for application, and organisational enablers (34, 35). Similar findings have been reported in the evaluation of health-focused MOOCs, where participants frequently report strong learning outcomes but encounter barriers to applying new knowledge within their professional contexts (36).

Participants also called for more detailed, practice-oriented content, including specific strategies, case-based scenarios, and guidance on implementation. This was particularly evident among participants with prior experience, who reported that some content was too basic and suggested the need for differentiated learning pathways. These findings highlight the challenge of designing workforce education programs that remain accessible to novice learners while providing sufficient depth for those with greater prior experience.

Barriers related to engagement and usability also emerged. Interactive components, particularly the discussion board, were consistently underutilised, with participants citing asynchronous timing, low activity, and lack of facilitation as limiting engagement. This aligns with previous research demonstrating that simply providing opportunities for interaction does not guarantee meaningful participation in online professional learning environments (37). Structured opportunities for interaction and facilitation may therefore be required to support engagement and peer learning (32). Technical issues, including platform usability and progress tracking, also affected engagement for a small minority of participants, highlighting the importance of reliable infrastructure and learning system design in digital workforce education (32).

These findings have implications for the future design and delivery of digital workforce training. Future iterations of the MOOC may benefit from incorporating facilitator-led or peer-moderated discussion activities, clearer prompts for interaction, and opportunities for learners to share practical experiences. Participants also highlighted the value of downloadable module summaries and supporting resources that can be revisited after course completion to support ongoing learning and application in professional practice. Routine quality assurance processes, adequate organisational support, reliable technical infrastructure, and ongoing investment in digital learning environments will also be important for effective implementation and a positive learner experience (28).

These findings suggest that MOOCs can support preparation of the public health workforce for digital health communication through accessible and flexible professional development. Beyond social media use, the course sought to build competencies in message framing, platform literacy, critical engagement with digital information environments, and recognition of commercial influences on health communication, capabilities that are increasingly important across digital health and virtual care settings. Maximising impact will require learning experiences that accommodate varying levels of prior experience and support application of learning in practice. More broadly, the findings highlight the potential of online learning to provide timely and flexible workforce development across public health, digital health, and virtual care settings. The digital communication environment has continued to evolve since the development of the MOOC, particularly through the rapid uptake of generative artificial intelligence tools. This may further increase the importance of competencies related to digital literacy, critical appraisal, content creation, and responding to mis/disinformation, reinforcing the need for workforce development initiatives that can adapt to rapidly changing digital environments (38, 39).

## 6 Strengths and limitations

The MOOC and its evaluation had a number of strengths. The MOOC was developed by a multidisciplinary team with expertise spanning academic and professional expertise in public health, health promotion, digital communication, social media, health behaviour change, learning design, and evaluation. The breadth of expertise contributed to the development of evidence-informed content that combined theoretical concepts with practical examples and real-world case studies. The course was specifically designed to be accessible to learners with varying levels of prior social media experience, using mixed media resources, expert-led content, and applied learning activities to support engagement and relevance to professional practice. In addition, the national reach of the evaluation enabled participation by public health and health promotion professionals across a range of jurisdictions, sectors, professional roles, and career stages. The use of both quantitative and open-ended survey responses collected at multiple time points provided complementary insights into participants' perceptions of relevance, learning outcomes, and early application of learning in professional practice.

However, there were some limitations. The data presented were derived from interim post-test and post-test survey responses within an ongoing evaluation and do not represent the final trial outcomes. Participants self-selected into the MOOC and evaluation, which may limit the generalisability of findings beyond Australian public health and health promotion professionals with an existing interest in digital communication. In addition, analyses were based on available responses at each survey wave and did not constitute a complete longitudinal cohort due to attrition, intermittent non-response, and incomplete linkage of survey responses across time points. Measures of practice change and application were self-reported and reflect early perceptions rather than sustained behaviour change. Longer-term impacts on practice and organisational outcomes will be examined in the full evaluation report, and future research would benefit from collecting information on reasons for non-response and attrition.

## 7 Conclusion

This study provides early evidence that a structured, online MOOC can support the development of digital communication capabilities among public health and health promotion professionals. Participants reported high levels of perceived relevance, knowledge gain, and skill development, particularly in relation to message framing, values-based communication, audience engagement, and understanding digital environments. Flexible delivery, expert-led content, and applied examples were identified as key facilitators of learning, while opportunities for interaction and differentiated learning pathways were identified as areas for improvement. Although participants reported positive impacts on professional practice, translation of learning into sustained practice change was less consistent, highlighting the challenges of moving from knowledge acquisition to implementation. These findings suggest that online professional learning can support preparation of the public health workforce for digital health communication and advocacy when sufficient attention is given to applied skill development, learner engagement, and opportunities for practice. As digital technologies continue to reshape health communication, public engagement, and service delivery, accessible workforce development initiatives remain important for building digital capability and confidence. Our ongoing evaluation of the MOOC will provide further insight into longer-term practice outcomes.

## Data Availability

The datasets presented in this article are not readily available because the ethical approval and participant consent arrangements do not permit data sharing.
